# Sir Isambard Owen

**Published:** 1927

**Authors:** 


### Obituary.

SIR ISAMBARD OWEN, D.C.L., M.D., F.R.C.P.

(*Vice-Chancellor of the University of Bristol*, 1909–24.)

We regret to have to record the sudden death in Paris, on January 14th, at the age of 76, of Sir Isambard Owen, formerly Vice-Chancellor of the University, and an honorary member of the Medico-Chirurgical Society. Sir Isambard came to Bristol in 1909 with an established reputation in University administration, for he had been Principal of the Armstrong College at Newcastle-on-Tyne, and Senior Deputy Chancellor of the University of Wales. The honour of knighthood, which he received in 1902, was a recognition of his work in and on behalf of the University of Wales.

The University of Bristol owed a great deal to his well-informed guidance in the early days. He was in touch with other Universities, and could voice the needs and views of Bristol at University conferences or with Government departments in such a way as to command attention. His experience as physician and dean to St. George's Hospital, enabled him to help the Faculty of Medicine with wise and sympathetic advice.

The foundation of a new University provided many problems which called for clear judgment and impartiality, both of which gifts Sir Isambard Owen possessed in a marked degree. In drafting statutes, ordinances and regulations, he was most adept, and on ceremonial occasions he proved a veritable master of pageantry. His speeches at public assemblies were admirable alike in style and in delivery.

A sympathetic obituary note in the *British Medical Journal* gave an excellent summary of his character :—

" Isambard Owen was a good friend and a charming companion. Slim and rather frail in appearance, he was capable of withstanding fatigue, whether physical, in his favourite pastime of cycling, or mental, when completing, under pressure, some of the laborious enquiries he undertook. He formed opinions only after full examination, but then held them tenaciously. His manner was always conciliatory, but opponents of a different temperament were often surprised how trenchant could be his criticisms, uttered in a quiet tone, and often illuminated by a flash of humour."

Among his other interests he numbered Freemasonry, and he attained to high honours in the Province of Bristol as Senior Grand Deacon. He had occupied the chair of St. Vincent Lodge as Worshipful Master in 1919.

Sir Isambard Owen married in 1905, and is survived by Lady Owen and two daughters, to whom we offer our deep sympathy.

